# Once upon a Time Oral Microbiota: A Cinderella or a Protagonist in Autism Spectrum Disorder?

**DOI:** 10.3390/metabo13121183

**Published:** 2023-12-05

**Authors:** Michele Mussap, Paola Beretta, Elena Esposito, Vassilios Fanos

**Affiliations:** 1 Laboratory Unit, Department of Surgical Sciences, AOU Cagliari, University of Cagliari, 09124 Cagliari, Italy; mumike153@gmail.com; 2 Neonatal Intensive Care Unit, Department of Surgical Sciences, AOU Cagliari, University of Cagliari, 09124 Cagliari, Italy; elena.esposito30@gmail.com (E.E.); vafanos@tiscali.it (V.F.)

**Keywords:** oral microbiota, autism spectrum disorder, gut–brain axis, children, gut microbiota, leaky gut, metabolomics

## Abstract

Autism spectrum disorder (ASD) is a neurodevelopmental disorder evolving over the lifetime of individuals. The oral and gut microbial ecosystems are closely connected to each other and the brain and are potentially involved in neurodevelopmental diseases. This narrative review aims to identify all the available evidence emerging from observational studies focused on the role of the oral microbiome in ASD. A literature search was conducted using PubMed and the Cochrane Library for relevant studies published over the last ten years. Overall, in autistic children, the oral microbiota is marked by the abundance of several microbial species belonging to the *Proteobacteria* phylum and by the depletion of species belonging to the *Bacteroidetes* phylum. In mouse models, the oral microbiota is marked by the abundance of the *Bacteroidetes* phylum. Oral dysbiosis in ASD induces changes in the human metabolome, with the overexpression of metabolites closely related to the pathogenesis of ASD, such as acetate, propionate, and indoles, together with the underexpression of butyrate, confirming the central role of tryptophan metabolism. The analysis of the literature evidences the close relationship between oral dysbiosis and autistic core symptoms; the rebuilding of the oral and gut ecosystems by probiotics may significantly contribute to mitigating the severity of ASD symptoms.

## 1. Introduction

Autism spectrum disorder (ASD) is an early-onset heterogeneous, multifactorial neurodevelopmental disorder evolving over the lifetime of the affected individuals [1]. ASD’s core symptoms are deficits in social communication and interaction, associated with restricted, repetitive, and sensory–motor behaviors and interests requiring lifelong support [2,3]. In patients with ASD, comorbidities are very frequent, especially gastrointestinal (GI) disease, the most common medical condition associated with ASD [4], periodontal diseases, and allergies [5,6]. Autism is also associated with several oropharyngeal impairments, including buccal sensory sensitivity, taste and texture reluctances, and salivary transcriptome alterations [7,8].

The etiopathogenesis of ASD cannot be ascribed to a single factor; rather, the ASD etiology encompasses a wide range of causative events acting upon a vulnerable genetic background. Causative events include rare de novo high-penetrance mutations and/or a mix of environmental and epigenetic factors [9].

Worldwide, it has been estimated that about one in 160 children has an ASD, corresponding to at least 78 million people [10]; however, this rate represents the mean of available data, and it must be considered with caution, given the wide data fluctuation across studies. In Europe, data referred to in 2019 reported over 28 million autistic people, corresponding to an age-standardized prevalence of 0.37%; incident cases were estimated to be 603,790 [11]. According to results reported by the Autism Spectrum Disorder in the European Union (ASDEU) 2015–2018, in Italy, the ASD incidence rates increased from 10% to 17% per year [12]. In the USA, one child (aged 8 years) in 54 is affected by ASD [13]. Several factors contribute to the increase in ASD epidemiology over time; the most relevant is the identification of mild forms. Further factors include the following: broadening of the autism concept and corresponding changes in diagnostic algorithms; early diagnosis, even under two years of age; the dramatic increase in environmental risk factors; and better ascertainment due to heightened awareness and improved detection and early diagnosis [14]. However, the increasing prevalence of autism may be overestimated since it is based on data from administrative records rather than community-based studies [15]. Indeed, data emerging from the general population and systematic case-finding community-based surveys report no significant change in autism prevalence rates in childhood [16]. Although the average age of ASD diagnosis remains around 4–5 years of age [17], the early diagnosis of autism may be performed in children aged 12–24 months; during this early lifetime, it is possible to identify specific signs and developmental history associated with ASD, including poor eye contact, lack of visual tracking, no orientation to name, few imitation skills, lack of social interest, and restricted language [18].

This review aims to identify all the available evidence emerging from the literature and specifically focuses on the elucidation of the role of the oral microbiome in children with ASD and the identification of distinctive microbial traits compared to neurotypical children.

### 1.1. Microbiota and Autism Spectrum Disorder

A growing body of evidence supports the direct association between autism and the microbiome [19]. On the one hand, several studies, mainly conducted in animal models, revealed significant differences in gut microbiome composition and diversity between autistic and neurotypical individuals [20], suggesting gut dysbiosis as a key co-factor in the pathogenesis of autism [21]. On the other hand, other studies postulated that autism induces several lifestyle changes, including dietary preferences, which, in turn, promote gut dysbiosis [22]. Indubitably, the imbalance of gut microbial communities, namely dysbiosis, exerts considerable effects on the gut–brain axis due to the close relationship between the gut microbiome and the enteric endocrine, immune, and nervous systems [23]. Beyond the gut microbial ecosystem, the second most abundant microbiota in humans harbors the oral cavity, which is considerably involved in human health and disease [24].

Although the terms microbiome and microbiota are often used interchangeably, they differ from each other; the former refers to the collection of genomes of all the microorganisms found in a given environment, and the latter encompasses all the living members forming the microbiome [25]. The human microbiome consists of trillions of microorganisms [26,27], including bacteria (microbiome), fungi (mycobiome), viruses (virobiome), archaea (archaeome), and eukaryotic parasites (protozoan parasites); the inter-individual variability in microbiome composition depends on several factors, such as dietary regimen, lifestyle, stress, socio-economic conditions, work environment, diseases, and many others [28]. Remarkably, the number of bacteria significantly exceeds that of any other taxa, and thus, the term microorganisms is commonly used to refer to bacteria only.

The imbalance of microbial ecosystems, namely dysbiosis, is associated with various diseases; this association is particularly crucial in diseases affecting the central nervous system (CNS) and represents the complex network called the microbiome–brain axis. Deciphering the dysregulation of the gut–brain axis is challenging to better understand the pathophysiology of mental disorders and to offer innovative models for approaching the diagnosis and management of neurodevelopmental disorders. Briefly, the gut–brain bidirectional interaction elicits a complex mechanism consisting of epigenetic changes, for example, the DNA methylation of genes known to be modulators of microglial activity or implicated in synaptic pruning. On the other hand, microbial dysbiosis alters mRNA splicing, including that of genes associated with autism [29]. As a consequence, many encoded proteins involved in synaptic transmissions could be altered, including brain-derived neurotrophic factors (BDNFs), a class of synaptic plasticity-associated proteins significantly increased in the blood of autistic individuals [30]. Alterations in the structure and/or function of proteins involved in synaptic transmissions lead to impairments in the neural circuits of the cerebral cortex [15].

### 1.2. Oral Microbiota

The oral cavity harbors approximately 50 to 100 billion bacteria and 600 microbial species, with different subsets colonizing distinct habitats [31]. These species belong to 185 genera and 12 phyla, namely *Firmicutes*, *Fusobacteria*, *Proteobacteria*, *Actinobacteria*, *Bacteroidetes*, *Chlamydiae*, *Chloroflexi*, *Spirochaetes*, *SR1*, *Synergistetes*, *Saccharibacteria (TM7)*, and *Gracilibacteria (GN02)*. Nearly 54% are formally called, 14% are unnamed but cultivated, and 32% are known only as uncultivated phylotypes [32]. The oral microbiome is located in a complex environment that includes distinct, small microbial habitats, like teeth, buccal mucosa, soft and hard palate, and the tongue; each of them creates a heterogeneous and peculiar ecosystem.

Oral dysbiosis leads to local and systemic alterations. The former consists of infections and inflammations, including dental caries and periodontal diseases. Systemic effects of oral dysbiosis may be detrimental for the development of non-communicable diseases, predominantly obesity, diabetes, cardiovascular disease, autoimmune disease, several types of cancer [33,34], and even adverse pregnancy outcomes [35]. Even though it is hard to define a causal link between oral dysbiosis and systemic disorders, the study of the oral microbiota may be considered a strategic tool for better understanding the origin and clinical outcome of various human diseases [36].

**Figure 1:**
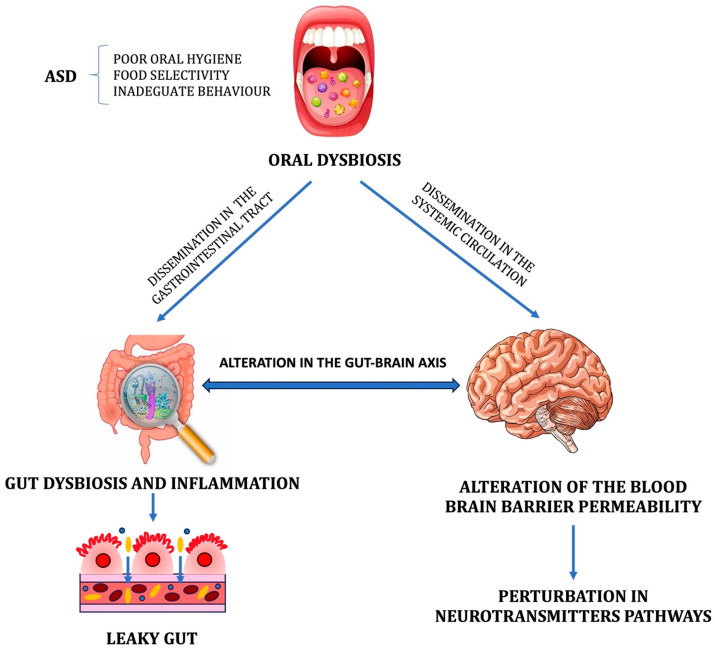
The potential impact of oral dysbiosis on the gut microbial ecosystem and blood–brain barrier (BBB). The resulting gut dysbiosis and inflammation significantly alter the gut–brain axis, while the altered permeability of the BBB promotes microbial brain contamination and the entry of toxic metabolites into the central nervous system, resulting in perturbations in the neurotransmitter pathways.

Similarly to the gut–brain axis, the oral microbiome may be involved in bidirectional crosstalk with the CNS [37]. It has been postulated that the oral microbial ecosystem may affect or contribute to the development and severity of neuropsychiatric diseases [38]. In particular, the translocation of specific bacterial taxa from the mouth to the bloodstream triggers many adverse mechanisms, including the systemic activation of pro-inflammatory cytokines, leading to so-called neuroinflammation, the colonization of pathogens in various organs and tissues, and the spread of toxic microbial metabolites [39]. The activation of these mechanisms leads to one of the most serious consequences: the loss of integrity of the blood–brain barrier (BBB). As a consequence, several toxic metabolites gain entry into the CNS and induce perturbations in the metabolic pathways involved in synaptic transmission (Figure 1). Even bacteria could colonize the CNS [40]. All the mentioned factors cause neuronal loss and synaptic deficits with the development of cognitive and behavioral impairment.

The oropharynx is innervated by five cranial nerves [41], including the pharyngeal branch of the vagus nerve, which enables the connection with enterochromaffin cells. Moreover, the trigeminal nerve, the fifth paired cranial nerve, connects the brain via the oral–brain axis [42]. In the olfactory tract, the olfactory nerve may act as a potential mediator for bacterial dissemination into the brain through the bloodstream, disrupting the BBB, perivascular spaces, or circumventricular organs [39]. This finding suggests that alterations in the microbial ecosystem of the oral cavity could impact the origin and severity of ASD [43]. Notably, ASD is marked by oropharyngeal abnormalities such as apraxia of speech and taste/texture aversions [44]. In addition, oral dysbiosis strongly impacts gut dysbiosis (Figure 1), with the oropharynx being the unique entry point into the GI tract. In particular, the oropharynx acts as a bridge between the oral and gut microbiomes [45]. Results emerging from the Human Microbiome Project [46] demonstrate that oral bacteria resemble 45% of stool bacteria, supporting the assumption of a close interaction between the gut and oral microbiomes. An experimental study demonstrated that after three hours from the inoculation of *Porphyromonas gingivalis* into the mouth, the pathogen was recognized in the ileum and after 16 hours in the colon [47].

Alterations in oral microbiota are not only caused by bacterial species but also by the overgrowth of fungi, viruses, and other microorganisms; the most frequently observed fungal overgrowth is *Candida* sp. Several studies have associated the overgrowth of this fungus with the pathophysiology of autism. [48,49,50]. This hypothesis is supported by the food selectivity often recognized in these children, who prefer foods rich in sugar; this habit creates an oral and intestinal environment conducive to overgrowth of *Candida* sp. and caries in general [51].

## 2. Methods

A literature search was conducted using PubMed, Scopus, and the Cochrane Library for relevant studies published from 2018 to 2023. Medical Subject Heading (MeSH) terms and the following free text terms were used: (“oral microbiome” OR “oral microbiota” OR “tongue microbiota” OR “gut-brain axis”) AND (pediatric OR child OR children) AND (“autism spectrum disorder”). We restricted the search to human studies published in English. The titles and abstracts of the retrieved studies were reviewed to exclude irrelevant studies. Then, two authors independently read the full texts of the remaining studies to assess their eligibility according to the inclusion criteria.

Eligibility criteria included any clinical study on the association between ASD and oral microbiota in children (e.g., cross-sectional, prospective longitudinal, case-control, randomized double-blind placebo-controlled trial, multicenter observational, community-based cohort, retrospective population-based cohort). Exclusion criteria included abstracts published in conference proceedings, book chapters, letters to the editor, guidelines, position papers, systematic reviews, meta-analyses, in vitro studies, and full-text articles written in languages other than English.

ASD was classified according to the *Diagnostic and Statistical Manual of Mental Disorders*, 5th Edition (DSM−5) [52]. The very small number of available articles in the databases hampered the application of the preferred reporting items for systematic reviews and meta-analyses (PRISMA) flow diagram [53]; we analyzed and discussed articles reporting data on the association between oral microbiota and ASD.

## 3. Results

Over the past decade, the human oral microbiota has raised the interest of research in autism, with the aim of improving the diagnosis, the utilization of diet supplementation with prebiotics and probiotics, and the application of individualized therapeutic protocols.

Poor oral health and hygiene, dental caries, and the lack of dental care are very frequently observed in autistic children [54], suggesting that ectopic transfer of pathogenic bacteria into the gut due to oral dysbiosis might explain the role of oral infections in the pathogenesis of ASD. Various factors contribute to poor oral health in ASD patients, including mouth infections, metabolic alterations caused by antipsychotic treatments, food selectivity, and lifestyle (for example, a high-sugar diet, the use of psychoactive medications, and inadequate oral and dental hygiene).

In patients with neurodevelopmental disorders, specific oral microbial fingerprints have been observed; this finding suggests that neurological disabilities could orchestrate the oral microbiome diversity and composition. This assumption is supported by the observation that the CNS can influence the oral microbiome in the same way it affects the gut microbiome [55].

Recently, a pre-clinical study using a mouse model found that oral dysbiosis is marked by the overgrowth of the *Streptococcus* genus in plaque and *Haemophilus* spp. combined with the depletion of helpful bacteria such as the *Prevotella* genus [56]. It is reasonable to assume that *Haemophilus parainfluenza*, a Gram-negative bacterium associated with oral diseases, and its metabolites could cross the altered BBB and negatively impact brain development, leading to cognitive impairment. The reduced microbial diversity in the gut ecosystem of autistic children was found to be equal to that in the oral cavity, with the significant prevalence of *Proteobacteria* and *Firmicutes* phyla and the depletion of *Bacteroidetes* and *Actinobacteria* phyla, confirming the dynamic interplay between the gut and oral microbiota [57]. In addition to this study developed on a mouse model, only four studies reported data on oral dysbiosis in children with ASD, marked by reduced microbial diversity and composition when compared with the oral microbiome of neurotypical (NT) children (Table 1).

The first study investigated alterations in the oral microbiome in children with ASD compared to NT children [58]. Overall, 32 autistic children (84% males) and 27 gender- and age-matched NT children (78% males) were recruited from primary schools. All the enrolled children provided salivary samples; 26 ASD children and 26 NT children provided samples collected from the teeth. Results obtained in dental samples of ASD children showed a significant reduction in alpha diversity; conversely, salivary samples showed no difference in alpha diversity between groups. The taxonomic analysis demonstrated that in both cohorts of children, *Firmicutes*, *Proteobacteria*, *Actinobacteria*, *Bacteroidetes*, and *Fusobacteria* were the most abundant phyla in both salivary and dental samples (overall 98.06% and 94.86%, respectively), with a higher predominance of *Proteobacteria* in children with ASD. In autistic children, the overgrowth of *Streptococcus* and *Haemophilus* genera in plaque and saliva, respectively, and the depletion of *Prevotella*, *Selenomonas*, *Porphyromonas*, *Actinomyces*, and *Fusobacterium* genera were observed. Interestingly, the *Rothia* genus was more abundant in dental samples and depleted in salivary samples of autistic children.

A second study was a cross-sectional, observational, case-control study enrolling one of the largest patient cohorts [59].

The study examined the oropharyngeal microbiome in 180 children with ASD (85% males), 106 NT children (60% males), and 60 children with non-autistic neurodevelopmental delay (DD) (70% males). Results indicated a higher rate of GI disorders in ASD and non-autistic DD children (22% and 20%, respectively) compared to NT children (3%) and a higher rate of immunologically mediated medication or food hypersensitivity reactions (21%) compared to the NT and DD cohorts (9% and 8%, respectively). The core oral microbiome included the *Proteobacteria* phylum, *Pasteurellaceae* and *Flavobacteriaceae* families, *Neisseria* and *Streptococcus* genera, *Streptococcus pneumonia*, *Streptococcus mitis*, *S. mitis B6*, *Streptococcus* sp. *oral taxon 064*, and *Gemella* sp. *oral taxon 928.* Overall, *Firmicutes* was the most abundant phylum, with *Lactobacillales* and *Bacillales* being the most prominent orders. No differences in alpha diversity were observed between groups, whereas significant differences were observed in beta diversity, with the NT children showing the highest rate of diversity between groups. The comparison between autistic and NT children showed the overgrowth of the *Plactomycetales* phylum and *Limnohabitans* genus and the depletion of the *Mucilaginibacter* and *Gemmata* genera, *Bacteroides vulgatus*, and *Ramlibacter tataouinensis*. On the other hand, the comparison between autistic and non-autistic DD children revealed the overgrowth of the *Brucella* genus and *Enterococcus faecalis* and the depletion of the Flavobacterium genus. No difference in bacterial phyla was observed between autistic children with GI disorders and those without GI disorders. However, the overgrowth of 25 taxa and the depletion of 3 taxa marked the oral microbiome of ASD children with GI disease.

A further interesting result was the evidence that alterations in the oral microbiome in ASD children imply differences in the RNA microbial profile targeting specific metabolic pathways in the oropharynx. Briefly, it was observed that there was an upregulation of microbial RNAs related to lysine catabolism in the oropharynx of autistic children, leading to an excess of glutamate. As is well known, glutamate is a key neurotransmitter involved in learning and memory [60], and glutamate excess results in excitotoxic effects in the CNS [61]. However, a direct relationship between oral dysbiosis and glutamate excess has not yet been demonstrated. The oral microbial RNA upregulation was also related to the overexpression of energy metabolism and carbon metabolism. These metabolic changes originating from oral dysbiosis represent a mechanism by which the interplay between host and microbiome may lead to abnormal social behaviors. Toxicological effects promoted by alterations in oral *Cyanobacteria* represent a second mechanism. The water-borne pathogens *Cyanobacteria* produce cyanotoxins; in autistic children, the overgrowth of oral *Cyanobacteria* generates an excess of cyanotoxins, inducing severe clinical signs and symptoms such as fever and GI disease.

The study by Kong X et al. reported the first comparative analysis between the gut and oral microbiota in autistic and NT children [62]. The intestinal colonization by oral bacteria through the ectopic transfer of pathogens (e.g., *P. gingivalis* in chronic periodontitis) promotes gut dysbiosis and inflammation, which progressively spread through the host. The aim was also to identify candidate microbial indexes/biomarkers for the diagnosis and monitoring of autism. Three tests were suggested as follows: the *Firmicutes/Bacteroidetes* ratio in the gut and the abundance of *Butyricimonas* and *Parvimonas* genera in the gut and saliva, respectively. The oral microbiota of autistic children was marked by the overgrowth of the *Bacilli* genus. It is unclear whether *Bacilli* genus overgrowth in the mouth and the gut of ASD patients may be due to environmental factors (e.g., diet) or whether *Bacilli* could migrate from the mouth to the gut. Nevertheless, evidence of gut colonization by oral microbial overgrowth may open new perspectives on the utilization of salivary microbial indexes/biomarkers to detect and confirm gut dysbiosis. Further evidence is the increased oral overgrowth of the *Bacilli* genus (*Firmicutes* phylum) both in the mouth and the gut of ASD children and in those with inflammatory bowel disease (IBD) [62]. Previous studies conducted in patients with IBD detected relevant amplifications in facultative anaerobic commensal gut bacteria belonging to the *Bacilli* genus; this finding supports the theory of a close relationship between *Bacilli* genus overgrowth and gut inflammation [63].

**Table 1:** Main characteristics and results of articles included in the review.

Year	Patients	Sample	Method	Oral Microbiome in ASD	Ref.
2022	15 mice inoculated with saliva from ASD donors (AOMO) 15 mice inoculated with saliva from TD donors (TOMO) 15 mice not receiving any microbes (CON)	Mice saliva and feces	16S rRNA	Alpha diversity decreased in the AOMO group as compared with the TOMO and CON groups in oral and fecal samples from mice Overgrowth (saliva): *Bacteroidetes* [G-7], *Bacteroidetes* [G-3], *Tannerella*, *Porphyromonas sp*. HMT, and unclassified *Bacteroidales* Depletion (feces): *Kytococcus* and *Desulfobulbus*	[56]
2018	32 ASD aged 7–14 y 27 HC aged 8–11 y	Saliva Gingival plaques from caries-free molars	16S rRNA	Microbial richness and diversity significantly lower in dental plaques Overgrowth: *Proteobacteria*, *Streptococcus* (in saliva), *Haemophilus* (in dental plaques) Depletion: *Actinobacteria* (in saliva), *Bacteroidetes* (in dental plaques), *Prevotella*, *Alloprevotella*, *Selenomonas*, *Actinomyces*, *Fusobacterium*, *Porphyromonas*	[58]
2018	180 ASD aged 53 ± 16 y 106 TD aged 43 ± 16 y 60 DD aged 50 ± 13 y	Saliva	Shotgun sequencing by NGS	Overgrowth (ASD vs. TD): *Limnohabitans*, *Planctomycetales* spp., *Cyanobacteria*, *Cellulomonas fimi ATCC 484* Overgrowth (ASD vs. DD): *Brucella*, *Enterococcus faecalis* Depletion (ASD vs. TD): *Bacteroides ovatus*, *Bacteroides vulgatus*, *Mucilaginibacter* sp. Depletion (ASD vs. DD): *Flavobacterium spp.*, *Chamaesiphon minutus PCC 6605*, *Pseudomonadaceae*	[59]
2019	20 ASD aged 7–21 y 19 NT aged 7–55 y	Saliva	16S rRNA	No significant difference in alpha and beta diversity Overgrowth: unspecified *Bacilli* genus, *Gemellaceae*, *Rickettsiales*, *Propionibacterium* Depletion: *Parvimonas*, *TM7 Saccharibacteria*	[62]
2021	25 ASD aged 9.2 ± 1.9 y 38 NT aged 10 ± 1.5 y	Tongue scrapping	16S rRNA	No significant difference in species richness, alpha and beta diversity Overgrowth: *Actinomyces odontolyticus*, *Actinomyces lingnae* Depletion: *Campylobacter concisus*, *Streptococcus vestibularis*, *Bergeyella sp. oral taxon 322*	[64]

Abbreviations: ASD, autism spectrum disorder; NT, neurotypical; TD, typically developing; DD, non-autistic developmental delay; NGS, next generation sequencing.

Given that *Firmicutes*, *Bacteroidetes*, and *Proteobacteria* are the most represented microbial phyla in the gut of autistic and non-autistic patients, it is reasonable to pursue the search for diagnostic tools unveiling dysbiosis, such as the ratio of Firmicutes/Bacteroidetes. This index may discriminate between autistic and NT individuals, being significantly influenced by the depletion of Bacteroidetes in autism. Unfortunately, data from the literature are often controversial, and several studies have reported a decrease in the ratio of Firmicutes/Bacteroidetes in autistic individuals. The analysis of oral microbiota can predict the level of *Bifidobacteria*, *Clostridiales*, and *Escherichia* genera in the gut; all of them are positively associated with ASD and GI disease.

A cross-sectional study investigated the microbiome of the tongue in 25 autistic children (16 males) and 38 NT controls (18 males) [64]; overall, 8 bacteria phyla, 51 genera, and 193 species were detected. The mean number of bacterial species and genera identified per subject was 132 and 39, respectively, ranging from 56 to 170 species and 20 to 47 genera. In both groups, approximately 99% of the sequences consisted of *Firmicutes*, *Actinobacteria*, *Proteobacteria*, *Fusobacteria*, and *Bacteroidetes* phyla. The most abundant species were *Haemophilus parainfluenzae*, *Rothia mucilaginosa*, and *Prevotella melanogenic*. Surprisingly, this study did not find significant differences in species richness, alpha diversity, and beta diversity between the tongue microbiome of autistic children and that of NT children. This finding may be related to the unique microbial environment of the tongue and the absence of significant differences in dental health between autistic and NT children enrolled in this study.

The previously mentioned study, conducted in a mouse model, investigated the role of the oral microbiota in the pathogenesis of ASD [56]. Qiao Y et al. transplanted oral microbiota from ASD and NT donors into an antibiotic-mediated microbiota-depleted mouse model. It was observed that the oral microbiota transferred from autistic donors induces abnormal behaviors mimicking ASD, such as restricted, repetitive, and stereotyped behaviors. Mice colonized by the oral microbiota of autistic donors showed significant differences in the microbial composition of the oral cavity and intestinal tract compared with mice receiving oral microbiota from NT donors and with those that did not receive any bacterial transfer. Alpha diversity was decreased in the group of mice receiving oral microbiota from the ASD donor (AOMO) as compared with the group of mice receiving oral microbiota from the NT donor (TOMO) and with that of mice not receiving any microbiome transplantation (CON), confirming differences between groups in microbiota composition.

In the AOMO group, the oral microbiome was depicted by the overgrowth of *Bacteroidetes [G-7]*, *Bacteroidetes [G-3]*, *Tannerella*, and unclassified *Bacteroidales*, whereas in the TOMO group, the oral microbiome was depicted by the overgrowth of *Kytococcus* and *Desulfobulbus* genera. In fecal samples, the most represented phyla were *Bacteroidetes*, *Proteobacteria*, and *Firmicutes.* In the AOMO group, the *Enterobacteriaceae* family and *Enterococcus* genus were more abundant; in the TOMO group, the *Porphyromonas* genus, *Bacteroidetes* phylum, and *Porphyromonadaceae* family were significantly enriched. The *Bacteroidales* order was significantly enriched in the ASD donor and AOMO mice.

*Bacteroidetes*, especially the *Porphyromonas* genus, have been identified as pathobionts, and a large body of literature has reported that these pathobionts contribute to periodontitis and cerebrovascular diseases through the release of virulence factors disrupting host immune homeostasis, such as lipopolysaccharides (LPSs) [55,65]. Specifically, pathobionts degrade dietary polysaccharides that generate short-chain fatty acids (SCFAs), such as butyric acid, propionic acid, and acetic acid. An excess of propionic acid in the brain of autistic subjects negatively impacts the metabolism of neurotransmitters by enhancing calcium-dependent serotonin and glutamate release [57,66].

Behavioral tests in recipient mice revealed significant differences in social deficits and repetitive behaviors. Compared with mice included in the TOMO and CON groups, mice belonging to the AOMO group spent a very long time interacting with an empty cup (*p* = 0.01); moreover, mice included in the AOMO group exhibited decreased exploratory behavior and social novelty with increased anxiety. Mice belonging to the TOMO group spent much more time interacting with an extra-family mouse than with the familiar one.

The RNA sequencing of the prefrontal cortex of recipient mice unveiled the upregulation of 375 genes; those encoding serotonin receptors and transporters were significantly upregulated in mice belonging to the AOMO group as compared with those belonging to the TOMO group. The evidence of a close correlation between the upregulation of these genes and the overgrowth of various bacterial species, including *Bacteroidetes [G-7]*, *bacterium HMT 911*, *Porphyromonas* spp., *Prevotella* spp., *Tannerella* spp., and *Erysipelotrichaceae [G-1]* spp., in the oral cavity of ASD donors and AOMO mice suggests a strong influence of oral dysbiosis on the overexpression of serotonin receptor genes.

## 4. Discussion and Conclusive Remarks

Since the oral cavity is the initial point of entry to the digestive and respiratory tract, it could be postulated that the accumulated evidence regarding the link between the intestinal microbial ecosystem and neurodevelopmental disorders boosts future studies related to the oral microbiota.

Studies in animal models and in humans demonstrated that oral pathogens migrate to the gut, inducing gut dysbiosis and leading to alterations in gut mucosal permeability; as a result, the consequent activation of the immune system promotes local and systemic inflammation [67]. The oral cavity can act as a reservoir for potential intestinal pathobionts that may exacerbate chronic intestinal disease [68]. For example, the overgrowth of Proteobacteria is associated with metabolic syndrome and IBD [69]. The microbiota–gut–brain axis is a bidirectional network that allows for the transfer of immune-active mediators, metabolites, toxins, neurotransmitters, and microorganisms from the gut to the brain and vice versa [70,71,72]. Routine dental interventions could cause transient bacteremia, and some pathogens may cross the BBB, reaching the brain, where they promote inflammation and alter neuroimmune activity. The bacterial migration from the mouth to the brain is facilitated by the loss of integrity of the BBB in ASD individuals due to microglia deterioration [73]. Recent studies suggest that the gut microbiota is involved in gut–brain communication, and gut microbes may play a role in modulating neural development. The aggregation of this knowledge supports the notion that the gut and probably the oral microbiota contribute to the development and severity of neurological and neuropsychiatric diseases [74].

Oral dysbiosis in ASD could be reflected by changes in the metabolic profile with the overexpression of metabolites closely related to the pathogenesis of ASD [75]. The increase in acetate and propionate, in conjunction with the decrease in butyrate and the microbial synthesis of indoles, confirms the central role of biochemical mechanisms in ASD and, in particular, the role of tryptophan metabolism [76]. Further metabolic alterations associated with ASD are the upregulation of 3-(3-hydroxyphenyl)-3-hydroxy propionic acid, 3-hydroxyphenyl acetic acid, and 3-hydroxyhippuric acid, indicating perturbations in phenylalanine metabolism [77]. The accumulation of specific metabolites (e.g., *p*-cresol) is due to the overgrowth of *C. difficile* and is associated with severe restricted and repetitive behaviors [78]. The considerable abundance of *C. difficile* in the guts of autistic individuals may reflect a pathogenic role for this pathogen. Whether metabolic changes result from oral dysbiosis in children with ASD remains to be determined.

The analysis of the articles included in this review may contribute to gaining awareness of the importance of the oral microbiota as a key co-factor for an earlier and more accurate diagnosis of ASD and for better management of the disease, especially to evaluate the response to therapeutic treatments. For example, deciphering the oral microbiome may contribute to the identification of subclinical or clinical subgroups of ASD patients with GI disease, autoimmune diseases, or inflammation. Recently, a salivary polyomic RNA measurement was proposed as a novel, non-invasive approach allowing for the accurate diagnosis of ASD [79].

Over the last few years, several studies have reported the beneficial effect of prebiotic consumption on oral dysbiosis [80]. For example, the regular consumption of glutamine, short-chain galacto-oligosaccharides, and long-chain fructo-oligosaccharides for approximately six weeks remodulates the structure of the oral microbiota [81]. On the other hand, robust evidence emerging from a large number of recent in vitro and in vivo studies suggests that a number of probiotics modulate oral bacterial species associated with mental disorders [80]. In particular, several probiotic strains, especially *Lactobacilli* and *Bifidobacterium* strains, have the ability to inhibit the growth of pathogens or to create a critical environment for their proliferation. For example, in an in vitro model, it was observed that *Lactobacillus rhamnosus* reduces the biofilm-forming capacity of *Fusobacterium nucleatum* [82]. These results have promoted the commercial availability of an increasing number of mouthwashes, oral sprays, and sticks containing probiotics, which are easily usable by patients.

In a mouse model of autistic animals, diet supplementation with the human commensal *Bacteroides fragilis* improved gut barrier integrity in maternal immune activation (MIA) offspring and ameliorated several core behavioral features of ASD [83]. The importance of probiotic supplementation during pregnancy to prevent autism-related behaviors in MIA offspring was recently confirmed in a further study on a mouse model [84]. *Bifidobacterium* (e.g., *B. longum*, *B. breve*, and *B. infantis*) and *Lactobacillus* (e.g., *L. helveticus* and *L. rhamnosus*) are probiotics commonly used in autistic individuals. These probiotics have demonstrated hopeful effects on abnormal behaviors, such as reductions in anxiety, depression, obsessive-compulsive disorder, and memory disturbances, including spatial and non-spatial memory [85].

Our review is affected by several limitations. The very small number of available studies on the oral microbiota in ASD hampered the application of the PRISMA workflow [53,86]. However, we conducted a careful analysis of the data reported in this group of studies, and thus, some preliminary conclusions may be obtained, despite the need for further studies to be conducted in larger patient cohorts. A further limitation is the lack of results on the effectiveness of therapeutic interventions in autistic subjects with oral dysbiosis. Clearly, this limit is closely related to the lack of this type of information in the selected articles. For example, the utilization of probiotics in autistic individuals may alleviate GI dysfunction and autism core symptoms by modulating the gut–brain and the oral–brain axes and reducing inflammation [87]. Future studies should explore whether metabolomics and microbiomics could predict the response to treatment with probiotic supplementation, applying individualized, tailored treatments to improve patients’ outcomes and minimize any adverse reactions.

## Abbreviations

ASD, autism spectrum disorder; GI, gastrointestinal; ASDEA, Autism Spectrum Disorder in the European Union; USA, United States of America; CNS, central nervous system; BDNF, brain-derived neurotrophic factor; BBB, blood–brain barrier; DSM–5, *Statistical Manual of Mental Disorders*, 5th edition; PRISMA, preferred reporting items for systematic reviews and meta-analyses; NT, neurotypical; DD, non-autistic developmental delay; IBD, inflammatory bowel disease; LPS, lipopolysaccharide; SCFA, short-chain fatty acid; MIA, maternal immune activation.
